# UFM1-Specific Ligase 1 Ligating Enzyme 1 Mediates Milk Protein and Fat Synthesis-Related Gene Expression via the JNK Signaling Pathway in Mouse Mammary Epithelial Cells

**DOI:** 10.1155/2020/4045674

**Published:** 2020-06-19

**Authors:** Meiqian Kuang, Min Yang, Lian Li, Chengmin Li, Genlin Wang

**Affiliations:** College of Animal Science and Technology, Nanjing Agricultural University, Nanjing, Jiangsu 210095, China

## Abstract

Ubiquitin-like modifier 1 ligating enzyme 1 (UFL1) has been characterized as a ubiquitin-like (Ubl) protein that affects a range of cellular processes across various pathways. In this study, mouse mammary epithelial cells (HC11 cell line) and UFL1 knockout (KO) mice were used to establish UFL1 knockdown models to explore the influence of UFL1 on milk protein and fat synthesis in the mouse mammary gland and the underlying mechanisms. This is the first study to show UFL1 localization in mouse mammary epithelial cells. UFL1 depletion by transfected UFL1 siRNA (siUFL1) caused aggravated apoptosis. In addition, UFL1 depletion suppressed milk protein synthesis-related protein level *in vivo* and *in vitro*. Conversely, ACACA and FASN expressions increased in UFL1-deficient mice. Moreover, UFL1 depletion increased triglyceride synthesis levels and inhibited the p-JNK expression. Importantly, the expression of proteins related to milk protein synthesis was decreased in JNK- and UFL1-deficient cells, whereas proteins related to milk fat synthesis showed the opposite trend, indicating that UFL1 affects milk protein and fat synthesis via the suppression of JNK activation. Overall, our findings indicate that UFL1 plays a key role in mammary milk and fat synthesis via JNK activation.

## 1. Introduction

Recently, the ubiquitin-fold modifier 1 (UFM1) conjugation system was characterized as a posttranslational modifier [1–4]. Ubiquitin-like modifier 1 ligating enzyme 1 (UFL1) is an important E3-ligating enzyme of the UFM1 conjugation system, which is expressed in multiple tissues including heart, liver, intestinal, and pancreatic tissues [5–9]. Further, it is well known that UFL1 is an indispensable component of this system given its involvement in various cellular processes, such as apoptosis [9, 10]. Targeted disruption of this protein is embryonically lethal, as it inhibits the embryonic development of the hematopoietic system 11.5 to 13.5 days after disruption in mice [9, 11].

The mammary gland is a critical organ for rearing neonatal offspring, as it provides them with milk, which is rich in nutrients, proteins, and fatty acids. Morphogenesis of the mammary glands during pregnancy and lactation permits milk production, and this process is regulated by various proteins, including posttranslational modification proteins, growth proteins, and transcription factors [12, 13]. Multiple physical and physiological cellular events (e.g., apoptosis, autophagy, stress response, and signal transduction) require posttranslational modifications in mammary epithelial cells during the synthesis of milk protein and fat [14, 15]. Therefore, further elucidation of the posttranslational protein-modifying system, such as UFL1, is critical to advance our understanding of various human organs and biological processes, particularly regarding mammary gland development during pregnancy and lactation.

It was reported that in human breast cells, UFM1 and UFL1 promote UFMylation of activating signal cointegrator 1 to prevent tumor growth via estrogen receptor-*α* [11]. Moreover, our previous study provided evidence that UFL1 alleviates inflammatory response and apoptosis induced by lipopolysaccharide (LPS) via the NF-*κ*B pathway in bovine mammary epithelial cells [16]. It has been further revealed that UFL1 plays a crucial role in maintaining homeostasis of mammary epithelial cells. Furthermore, a research found that UFL1 deficiency results in intestinal issues by causing a reduction in the numbers of paneth and goblet cells, which are critical cells of the intestine, and by altering the intestinal microbial environment constantly [7]. Evidence further suggested that UFL1 loss affects constitutive amylase levels in the exocrine pancreas, thus impairing pancreatic function [5]. Given that the mammary glands, intestines, and pancreas are secretory organs, it has been suggested that UFL1 plays important roles in the function of these types of organs.

c-Jun N-terminal kinase (JNK) further plays a crucial role in various cellular processes in the mammary glands. An important evidence has suggested that JNK is a fundamental regulator of mammary morphogenesis as it enhances epithelial cell death, thus contributing to effective mammary involution [17]. UFM1-binding protein 1 (UFBP1) is another member of the UFM1 system and its deficiency results in JNK accumulation, indicating that UFBP1 interaction with JNK is required for erythroid development [18]. Despite extensive research regarding other proteins involved in the UFM1 system, as well as the importance of UFL1 in mastitis and breast cancer, our knowledge of UFL1 function in the mammary gland requires further elucidation. Therefore, this study is aimed at further investigating the UFL1 function. We assumed that by affecting milk protein and fat synthesis in mammary epithelial cells via JNK suppression, UFL1 plays a critical role in mammary morphogenesis.

## 2. Materials and Methods

### 2.1. Ethical Statement

All procedures involving animals were carried out in accordance with the Guide for the Care and Use of Laboratory Animals provided by the Institutional Animal Care and Use Committee of Nanjing Agricultural University. All female mice used in this research were cared for in designated pathogen-free facilities.

### 2.2. Cell Culture

Mouse mammary epithelium cells (HC11 cell line) were purchased from the American Type Culture Collection. Cells were cultured in Roswell Park Memorial Institute (RPMI) 1640 medium (Gibco, USA) supplemented with 10% fetal bovine serum (FBS; Grand island, NY, U.S.A.) and 1% antibiotic (penicillin and streptomycin; Sigma-Aldrich, St. Louis, MO, U.S.A.). During this process, all cells were maintained at 37°C and 5% CO_2_.

### 2.3. Generation of UFL1 Knockout Mice

UFL1 knockout (KO) mice were provided by Dr. Honglin Li (Georgia Regents University, Augusta, GA, USA), and they were generated according to previously described methodologies [5, 19]. Adult female UFL1 KO mice were injected with tamoxifen (20 mg/mL in corn oil) by intraperitoneal route for five days. Seven days after the injection period, mice were sacrificed to collect mammary tissues after weighing the body and mammary weights. The efficiency of UFL1 knockout in mice was tested using western blot and qPCR.

### 2.4. Cell Transfection and Treatment

UFL1 siRNA (siUFL1) was designed using the following sequence: 5′-GGAUCCGUCAAGCGAUGAATT-3′ (GenePharma, China). siUFL1 was then diluted with diethylpyrocarbonate-treated water. For transient transfection, HC11 cells were plated in 6-well plates and subsequently infected with siUFL1 or control siRNA using Lipofectamine 2000 reagent (Invitrogen, Carlsbad, CA), according to the manufacturer's instructions. Only cells that had grown to 60%–80% of their final size were transfected with siUFL1 or control siRNA for 4 h in a reduced serum medium. After incubation, reduced serum medium containing the siRNA complexes was replaced with fresh medium until cells were collected for analysis. A JNK inhibitor, SP600125 (catalog number: 8177), was purchased from Cell Signaling Technology, dissolved in dimethyl sulfoxide (DMSO), and then used to treat cells for 2 h.

### 2.5. Immunofluorescence

HC11 cells were plated on 24-well plates (500 *μ*L RPMI 1640 supplemented with 10% FBS at 37°C and 5% CO_2_). HC11 cells were then fixed in 4% paraformaldehyde for 20 min and permeabilized with 0.5% Triton-X-100 (T9284, Sigma-Aldrich) for 30 min at room temperature. After cells were washed with PBS three times, they were blocked with 5% BSA (A4737, Sigma-Aldrich, USA) in PBS for 1 h at room temperature. Samples were then incubated with anti-UFL1 at 4°C overnight. Cells were washed using PBS and subsequently incubated with conjugated goat anti-rabbit IgG (1 : 400) in the dark and at room temperature for 2 h. Finally, cell nuclei were counterstained with DAPI (D8417, Sigma Aldrich) for 15 min and then mounted on glass slides. Cells were analyzed and photographed using a confocal laser scanning microscope (Zeiss LSM700 META). All experiments were independently carried out 3 times, and the information regarding the antibodies are summarized in Table 1.

### 2.6. Nuclear Protein Extraction

Nuclear and cytoplasmic proteins were extracted using an extraction kit (P0027, Beyotime Biotechnology) following the manufacturer's protocol. After washing the cells three times with PBS, they were incubated with 250 *μ*L cytoplasmic mix buffer for 30 min and then vortexed (three 15 s cycles). The lysates were then centrifuged for 10 min at 12000 × *g*, 4°C, and the resulting supernatants were cytoplasmic proteins. The pellets were resuspended in 50 *μ*L nuclear extraction buffer for 30 min and vortexed three times for 20 s. The nuclear lysates were centrifuged for 10 min at 12000 × *g*, 4°C, and the resulting supernatant was nuclear proteins.

### 2.7. Western Blot Analysis

The effective concentrations of siULL1 and JNK inhibitor were selected for exploring the role of UFL1 in the process of milk protein and fat synthesis. siUFL1 was transfected by Lipofectamine 2000 reagent. The concentration of 20 *μ*M of SP600125 used in the starvation medium for 2 h suppressed the JNK expression. After SP600125 treatment, proteins from the treated samples were collected and analyzed by western blotting. Cells were lysed in 200 *μ*L lysis buffer containing RIPA buffer (P0013B; Beyotime Biotechnology) and PMSF buffer (ST506, Beyotime Biotechnology). Protein concentrations were determined using the BSA assay kit (P0010; Beyotime Biotechnology). Equal amounts of protein samples (30–60 *μ*g) were separated using 12% SDS-PAGE and electrotransferred onto PVDF membranes (FFP39, Beyotime Biotechnology). Membranes were blocked with 5% free-fat milk for 2 h at room temperature and subsequently incubated with primary antibodies at 4°C overnight (Table 1). After three washes with PBS, membranes were subsequently rinsed with TBST for 5 min and incubated with secondary antibodies for 2 h at room temperature. The membranes were visualized using LAS-4000 (Fujifilm, Tokyo, Japan).

### 2.8. RNA Extraction and qPCR

RNA extraction and qPCR were performed as described in previous studies [20, 21]. Total RNA was extracted from target samples using TRIzol Reagent (Invitrogen). The concentration and purity of RNA were detected by spectrophotometer (NanoDrop Technologies, USA). Then, 500 ng cDNA was reverse transcribed from the RNA using a reverse transcription reagent kit (Takara, China). qPCR was used to analyze the expression of mRNA by SYBR green plus reagent kit (Roche, Swiss). All data were analyzed by 2^−*ΔΔ*Ct^ method, and *GAPDH* was used as the reference gene. The primers used are shown in Table 2.

### 2.9. Cell Viability Analysis

Cell viability analysis was conducted using CCK-8 assay kit (C0037, Beyotime Biotechnology). Cells were seeded at a density of 1 × 10^3^ cells/mL in each well of the 96-well plate. After cell treatment, 10 *μ*L of CCK-8 was added into each well for 4 h at 37°C. Finally, cell viability was tested at 450 nm by a microplate reader (Thermo Fisher Scientific). Each experiment was performed independently 3 times.

### 2.10. Flow Cytometry Analysis

Cells were seeded in 6-well plates and then transfected with siUFL1 for 72 h. Cells were collected and analyzed from the plate using FITC Annexin V Apoptosis Detection Kit I (BD, USA), according to the manufacturer's instructions. Briefly, cells were centrifuged and then suspended in 500 *μ*L of binding buffer. Cells were incubated with propidium iodide and Annexin V at room temperature in a dark room for 15 min. Fluorescence intensity was analyzed by flow cytometer with FACS Calibur (BD Biosciences, Bedford, MA). FlowJo 10 (Stanford University, USA) was used for data analyses.

### 2.11. TUNEL Assay

Apoptosis rates were determined using a TUNEL apoptosis assay kit (C1086, Beyotime Biotechnology). Cells were fixed in 4% paraformaldehyde buffer and permeabilized with 0.3% Triton-X-100 for 30 min at 37°C. Finally, cells were photographed using a confocal laser scanning microscope (Zeiss LSM700 META).

### 2.12. Histological Analysis

Mammary tissues were isolated from postpartum UFL1 KO mice and fixed in 4% paraformaldehyde buffer for 12 h at room temperature. After being embedded in paraffin, all sections were processed in xylene and a series of graded ethanol solutions for deparaffinization and rehydration. Finally, sections were stained with hematoxylin and eosin solutions and then mounted on slides with glass coverslips. Tissues were then photographed using a microscope (Nikon, Tokyo, Japan).

### 2.13. Detection of JNK Activity by ELISA

JNK activity in each sample was assessed using a JNK activity assay kit by ELISA (Meimian, China). Samples were lysed in PBS and then centrifuged at 12000 × *g* and 4°C for 15 min, according to the manufacturer's instruction. Finally, the density of each sample was immediately determined using a microplate reader (Thermo Fisher Scientific) at 450 nm.

### 2.14. Triglyceride Assay

Triglyceride level was determined using a triglyceride assay kit (A110-1-1, Jiancheng, China), according to the manufacturer's instruction. All samples were lysed with PBS and then centrifuged at 4°C for 10 min. Finally, all samples were measured at 510 nm using a microplate reader (Thermo Fisher Scientific).

### 2.15. Statistical Analysis

Statistical analyses were conducted using GraphPad Prism Software (GraphPad Prism Software Inc., San Diego, CA, USA). Data were expressed as mean ± SEM of three biological replicates. All results were compared by conducting *t*-tests or one-way analysis of variance (ANOVA) followed by Tukey's test. *P* values < 0.05 were considered statistically significant.

## 3. Results

### 3.1. Subcellular Expression of UFL1 in Mammary Epithelial Cells

**Figure 1:**
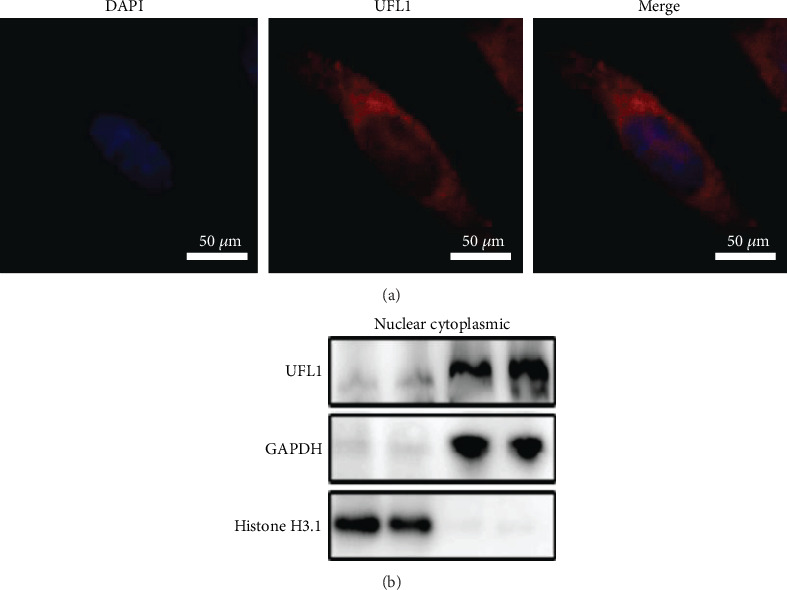
UFL1 expressed in mammary epithelial cells. (a) Expression of UFL1 was suggested by red fluorescence in HC11 cells. Scale bar = 50 *μ*m. (b) Western blot was performed to examine the expression of UFL1 in the nucleus and cytoplasm of HC11 cells.

To investigate the UFL1 function in mammary epithelial cells, we explored UFL1 localization in mouse mammary epithelial cells (HC11 cell line). Using immunofluorescence staining, we examined UFL1 expression in HC11 cells and found that UFL1 was indeed expressed in these cells (Figure 1(a)). To further examine UFL1 expression patterns in the nucleus and cytoplasm of HC11 cells, we isolated the nuclei and cytoplasm from these cells. Western blot analysis revealed that UFL1 expression in the nucleus was higher than that in the cytoplasm of HC11 cells (Figure 1(b)). Overall, these results confirmed the UFL1 expression in mammary epithelial cells.

### 3.2. Establishment of UFL1 Knockdown In Vivo and In Vitro

**Figure 2:**
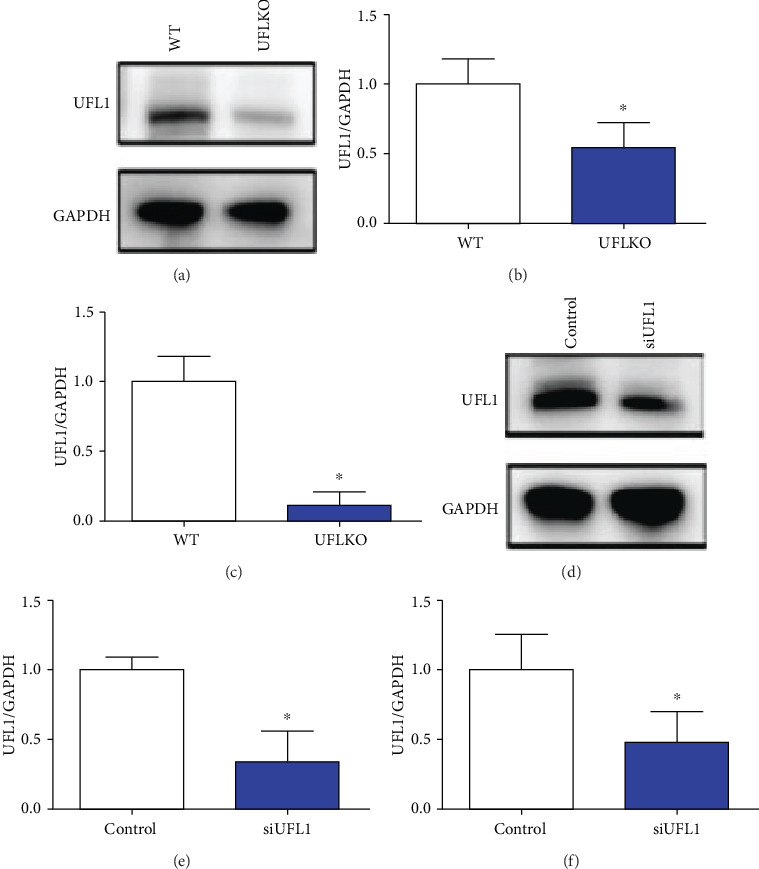
The efficiency of UFL1 in the mouse mammary. In this research, UFL1 KO mice and siUFL1 were used for the established UFL1 deficiency model. (a–c) The mammary was collected from UFL1 pregnant KO mice. (a, b) The expression of UFL1 was determined by western blot in UFL1 KO mice. (c) Relative expressions of UFL1 were tested by qPCR in UFL1 KO mice. (d–f) HC11 cells were transfected with UFL1 siRNA or control. (d, e) Using western blot for the expression of UFL1 or GAPDH in cell lysates. (f) Relative expressions of UFL1 were confirmed by qPCR in HC11 cells, followed UFL1 siRNA transduced. All experiments were tested three times. Graphs were expressed as the means ± SEM of 3 independent experiments. ^∗^ indicates significant difference (*P* < 0.05).

We found that, in UFL1 KO mice, protein and mRNA levels of UFL1 were significantly lower than those in the wild-type (WT) control (*P* < 0.05, Figures 2(a)–2(c)). In this study, a UFL1 sequence (siUFL1) was generated for *in vitro* experiments. Similarly, in our *in vitro* study, we found that the UFL1 mRNA and protein levels were significantly decreased compared with their expression in the control (*P* < 0.05, Figures 2(d)–2(f)).

### 3.3. UFL1 Deletion Affects Homeostasis in the Mammary

**Figure 3:**
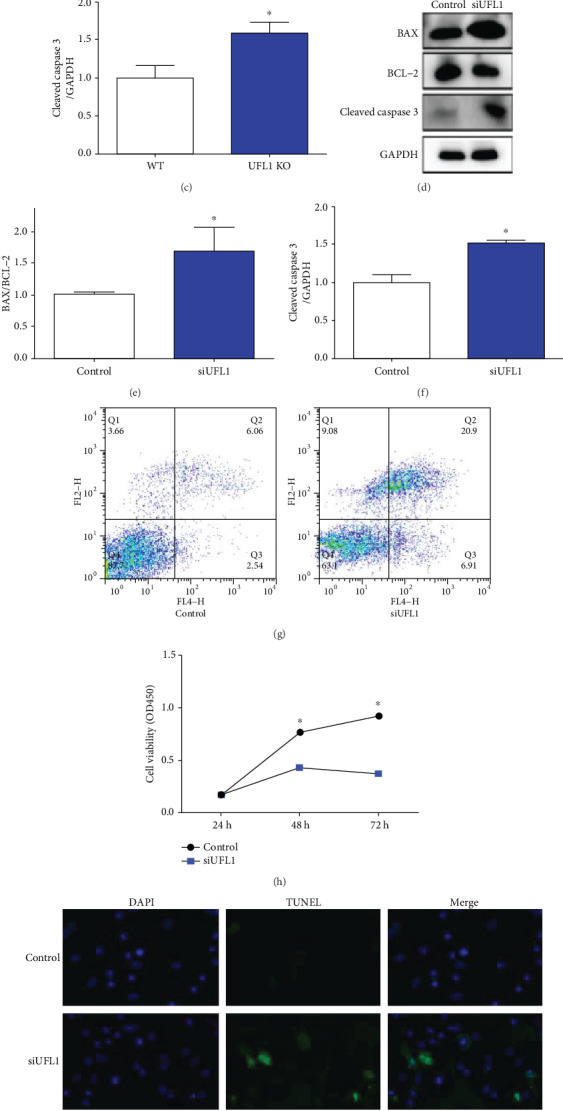
Effect UFL1 on proliferation and apoptosis in the mammary gland. (a–c) Mammary tissues were harvested from UFL1 KO mice after tamoxifen intraperitoneal treatment. (a) Samples were lysed, and the expressions of BAX, BCL and cleaved caspase 3 were analyzed by western blot. The relative intensity of (a) was plotted in (b) and (c). (d–i) HC11 cells were transfected with siUFL1 or control before being harvested. (d) Western blot analysis of process apoptosis-related proteins BAX, BCL, and cleaved caspase 3 levels. The relative intensity of (d) was plotted in (e) and (f). (g) HC11 cells transfected with UFL1 siRNA were stained with annexin and PI tested to flow cytometry followed and then quantification of apoptosis ratio. (h) Cell viability was detected by CCK-8 analysis. (I) TUNEL analysis was used for determining the apoptosis of HC11 cells followed by UFL1 siRNA treatment. TUNEL positive was shown as green fluorescence, and nuclei were stained with DAPI as blue fluorescence. Scale bar : 20 *μ*m. Results were analyzed by means ± SEM. ^∗^ explores significant difference (*P* < 0.05). N.S. indicates no significant difference (*P* > 0.05).

To confirm the influence of UFL1 on apoptosis, we analyzed the expression of apoptosis-associated proteins (BAX, BCL, and cleaved caspase 3) using western blot analysis *in vivo* and *in vitro*. Our *in vivo* results revealed that the WT, BAX, and cleaved caspase 3 levels in UFL1 KO mice were significantly higher, whereas BCL levels showed the opposite trend (*P* < 0.05, Figures 3(a)–3(c)). Similarly, BAX and cleaved caspase 3 expression levels were significantly increased in the siUFL1 group compared with those in the control (*P* < 0.05, Figures 3(e) and 3(f)). Alternatively, the BCL level was significantly decreased in the siUFL1-treated cells compared with that in the control group (*P* < 0.05). Furthermore, after siUFL1 transfection, apoptosis rate significantly decreased compared with that of the control group (*P* < 0.05, Figure 3(g)). Using CCK-8 proliferation assays, we revealed that cell viability under the siUFL1 treatment was significantly decreased in a time-dependent manner. We further found that cell viability did not significantly different between the siUFL1-treated and control groups at 24 h (*P* > 0.05, Figure 3(h)). However, HC11 cell viability decreased in cells transfected with siUFL1 at 48 h and 72 h compared with the control group (*P* < 0.05.). Our TUNEL assays further confirmed that UFL1 depletion disturbed cellular homeostasis given that the apoptosis level (green fluorescence) significantly increased following treatment of HC11 cells (Figure 3(i)). These results indicated that UFL1 disruption activated apoptosis in the mammary gland. In addition, to further determine the function of UFL1 on cell proliferation, we analyzed the expression of Cyclin D1 by western blot. We found that the protein level of UFL1 was significantly decreased in the disruption of UFL1 expression *in vivo* and *in vitro* works (*P* < 0.05, Supplement Figure 1).

### 3.4. UFL1 Disruption Does Not Affect Normal Mouse Mammary Morphology

**Figure 4:**
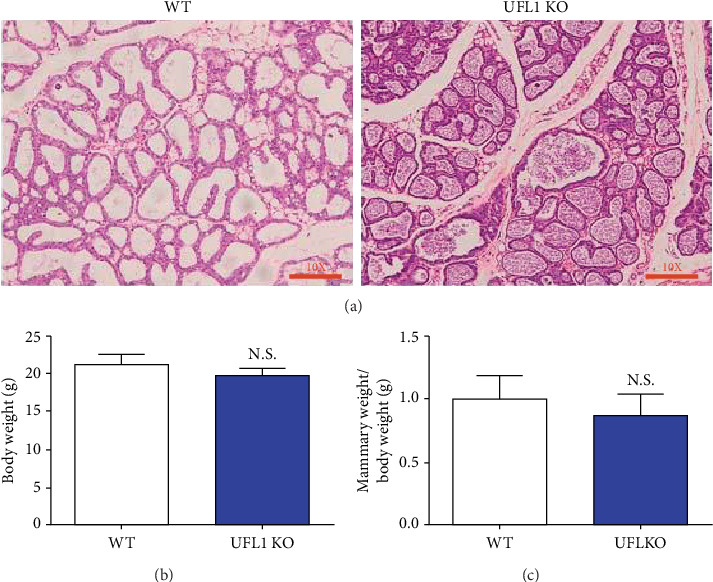
Deficiency of UFL1 does not affect morphology of the mammary gland. UFL1 KO mice in lactation were treated with tamoxifen, after 7 days, mammary tissues were collected. (a) Histologic observations of the mammary from UFL1 KO mice and WT mice. Bar = 20 *μ*m. The body weight (b), mammary weight (c), and relative mammary weight were analyzed in units of g, g, and g/g. The relative mammary weight was measured by dividing the mammary weight by the body weight. Data were represented with mean ± SEM by *t*-test (*n* = 3). ^∗^ denotes significant differences (*P* < 0.05).

Given that UFL1 was indeed expressed in mammary epithelial cells, we assessed whether UFL1 deficiency altered mammary gland weight and morphology. Hematoxylin-eosin staining in UFL1 KO and WT mice yielded similar results, wherein both mice exhibited normal morphology of the alveolar walls and microbial environments (Figure 4(a)). Compared with WT mice, body weight, mammary weight, and relative mammary weight did not significantly differ in the UFL1 KO mice (Figures 4(b) and 4(c)).

### 3.5. Loss of UFL1 Regulates Milk Protein and Fat Synthesis

**Figure 5:**
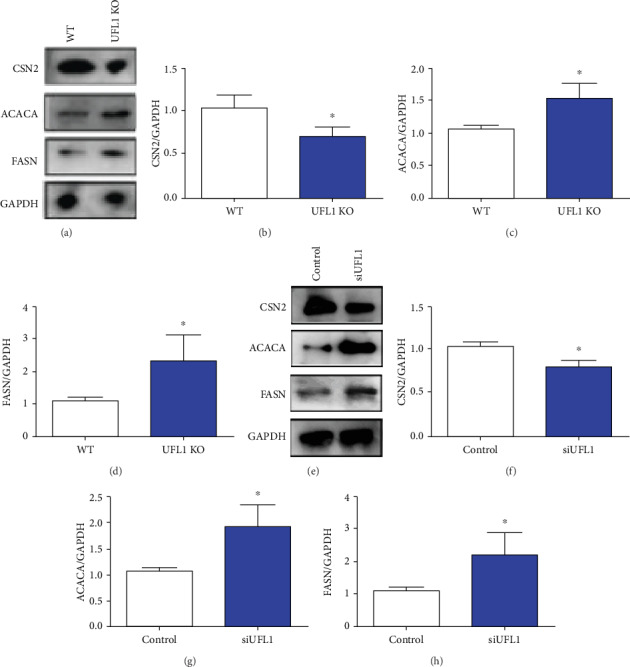
Deficiency of UFL1 affects milk protein synthesis in the mammary. (a–d) The lactation of the mammary gland was collected from UFL1 KO mice and WT mice as previously described. (a) After samples were harvested, western blot was used for analyzing milk protein synthesis-related proteins CSN2, ACACA, and FASN levels. The relative intensity of (a) was plotted in (b–d). (e–h) HC11 cells were transfected with control siRNA and vehicle (control) as previously described. (e) The levels of CSN2, ACACA, and FASN were determined by western blot. The relative intensity of (f) was plotted in (f–h). Data were analyzed by *t*-test with representative of 3 times independent experiments. Asterisks indicate significant difference (*P* < 0.05).

To assess UFL1 significance in milk protein and fat synthesis, we determined the levels of proteins related to these processes in UFL1-deficient mammary epithelial cells *in vivo* and *in vitro* using western blot analysis. CSN2 is an essential protein involved in milk protein synthesis [22]. Our results revealed that UFL1-deficient mice showed significant suppression of expression of CSN2 (*P* < 0.05, Figures 5(a) and 5(b)). In contrast, ACACA and FASN levels increased as a result of UFL1 deficiency. To further understand and validate this effect of UFL1 deficiency, we assessed the expression of these proteins under our *in vitro* experimental conditions. Consistent with the above, we found that CSN2 expression increased following UFL1 loss, but the ACACA and FASN levels also significantly increased.

**Figure 6:**
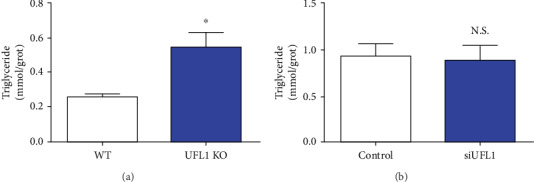
Triglyceride levels were elevated in the mammary from UFL1 KO mice. The level of triglyceride was detected by a triglyceride kit. (a) The level of triglyceride in mammary tissues was collected from UFL1 KO mice. (b) The level of triglyceride in cells was collected from transfected siUFL1. Graphs show mean ± SEM. ^∗^ denotes *P* < 0.05. n.s. indicates no significant difference.

To further elucidate UFL1 regulation of milk fat synthesis, triglyceride levels were determined. Triglyceride levels were evidently increased in UFL1 KO mice compared with those in WT mice (*P* < 0.05, Figure 6(a)). Furthermore, siUFL1-treated cells did not exhibit significant upregulation of triglyceride synthesis compared with the control group (*P* > 0.05, Figure 6(b)). Overall, these results suggest that UFL1 deficiency upregulates triglyceride synthesis and thus elevates ACACA and FASN protein levels in the mouse mammary gland.

### 3.6. UFL1 Depletion Suppresses JNK Activation

**Figure 7:**
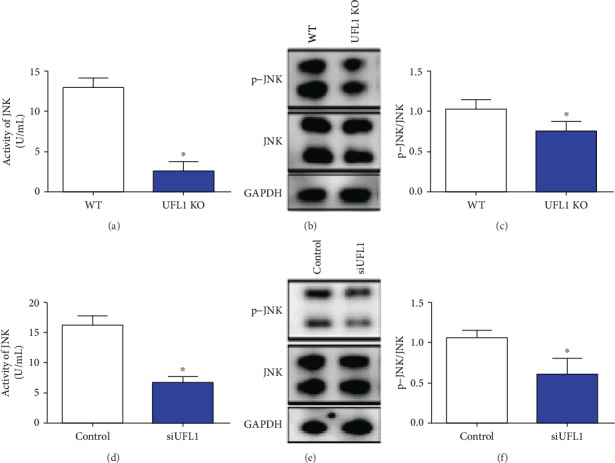
UFL1 suppressed the activation of JNK. (a–c) Mammary gland tissues were collected from UFL1 KO mice after tamoxifen intraperitoneal treatment. (a) The activity of JNK was tested by ELISA using a JNK activity assay kit. (b, c) The abundances of p-JNK and JNK proteins from mammary tissues of UFL1 KO mice were analyzed by western blot determined. (d–f) Cells were transfected with control siRNA or UFL1 siRNA for 72 h. (d) After treatment as previously described, cells were lysed in PBS, and then the activity of JNK was expressed by the JNK activity assay kit. (e, f) The levels of p-JNK and JNK were detected by western blot. The relative intensity was p-JNK relative to the JNK level. Bars were expressed as the mean ± SEM of the data. ^∗^ indicates significant difference (*P* < 0.05).

It has been reported that the c-Jun NH2-terminal kinase (JNK) is involved in epithelial organ morphogenesis and thus plays important roles in maintaining epithelial cell homeostasis. We hypothesized that UFL1 deficiency affects JNK activation. As illustrated in Figures 7(a) and 7(d), JNK activation, detected by ELISA kit, was significantly inhibited by UFL1 deficiency both *in vivo* and *in vitro* (*P* < 0.05). In addition, we used western blot analysis to calculate the normalized values of p-JNK relative to JNK and thus determine the expression levels of p-JNK and JNK. As shown in Figures 7(b) and 7(c), p-JNK expression was significantly lower (~3-fold) in UFL1 KO mice compared with that in WT mice (*P* < 0.05). We further revealed that p-JNK expression levels were also significantly lower in HC11 cells in response to UFL1 knockdown at 72 h (*P* < 0.05, Figures 7(e) and 7(f)). These results confirmed that UFL1 plays an important role in suppressing JNK activation.

### 3.7. Effect of JNK on Milk Biosynthesis in UFL1-Deficient Cells

**Figure 8:**
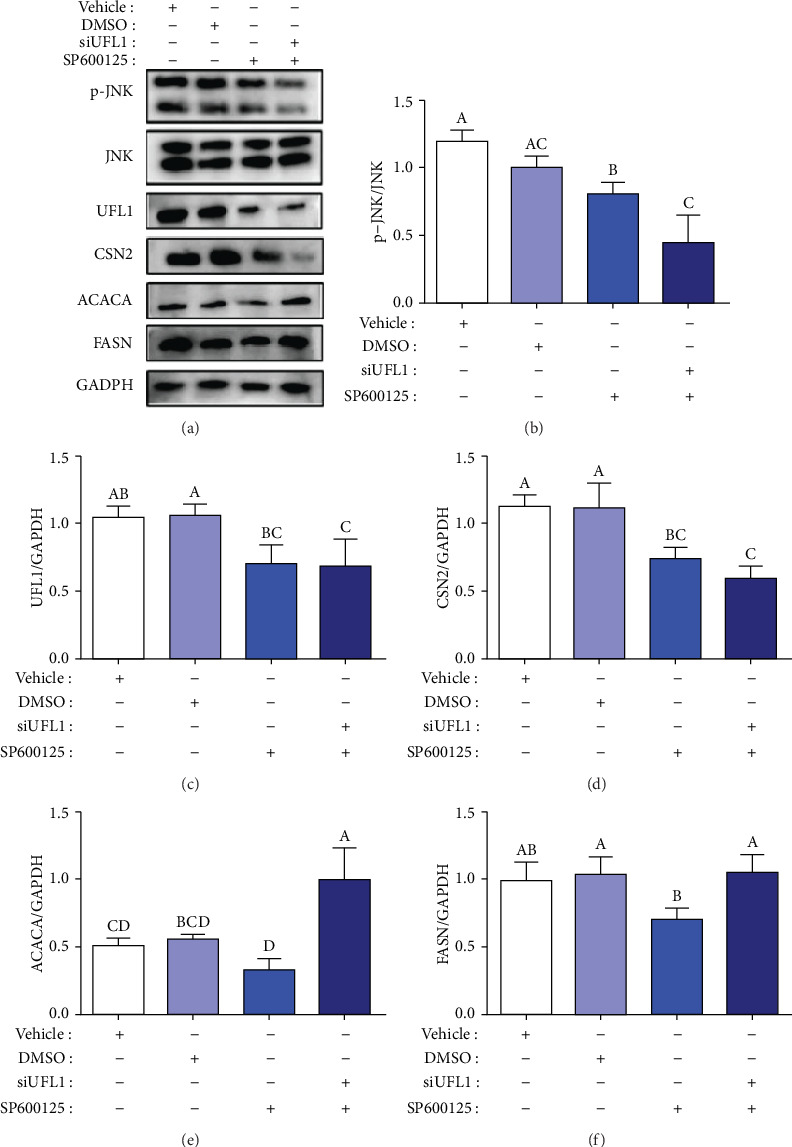
Effects of JUK inhibitor on milk protein and fat synthesis in UFL1 deficiency of HC11 cells. (a) The milk protein and fat synthesis-related proteins (p-JNK, JNK, UFL1, CSN2, ACACA, and FASN) were detected by western blot following treatment with SP600125 (an inhibitor of JNK, 20 *μ*Μ) alone or with siUFL1. The relativity of p-JNK, JNK, UFL1, CSN2, ACACA, and FASN was plotted in (b–f). Data were presented by one-way ANOVA with representative of 3 times independent experiments. Different letters indicate significant difference (*P* < 0.05).

We further assumed that UFL1 deficiency affects the levels of proteins related to milk proteins and fat synthesis via suppression of JNK activation. We used an inhibitor of JNK, SP600125, in the current study. Following SP600125 alone treatment, p-JNK and UFL1 levels were dramatically reduced compared with those in the control cells and DMSO-treated cells (*P* < 0.05, Figures 8(a) and 8(b)). Moreover, CSN2 expression decreased by approximately 38% (*P* < 0.05, Figure 8(d)), when treated with SP600125. Further, compared with the control, UFL1 and p-JNK levels were significantly reduced in cells cotreated with siUFL1 and SP600125 (*P* < 0.05), whereas the expression of CSN2 decreased by approximately 55% (*P* < 0.05, Figure 8(d)), compared with the control cells. However, CSN2 expression did not significantly differ when cells were treated with SP600125 alone, compared with that after co-treatment with SP600125 and siUFL1 (*P* < 0.05, Figure 8(d)). These results indicate that UFL1 negatively affects CSN2 expression via JNK suppression.

We also analyzed ACACA and FASN expression to further investigate whether UFL1 regulates milk fat synthesis via JNK suppression. Western blot analysis indicated that cells treated with SP600125 alone exhibited significantly lower ACACA and FASN expression levels compared with those expressed by the control and DMSO-treated cells (*P* < 0.05, Figures 8(e) and 8(f)). Conversely, compared with the control cells, ACACA expression dramatically increased in the siUFL1 and SP600125 cotreated cells (*P* < 0.05). Further, compared with control and DMSO-treated cells, FASN expression did not remarkably change in response to siUFL1 and SP600125 cotreatment (*P* > 0.05). These results indicate that UFL1 is a positive regulator of milk fat synthesis and has a negative effect on the expression of milk proteins.

## 4. Discussion

Mammary epithelial cells play critical roles in various cellular processes and in maintaining the physiology and homeostasis of the mammary gland. In the present study, we provide new insights into the regulation of UFL1 in response to cellular events by UFL1-mediated suppression of JNK activation in mouse mammary epithelial cells. We found that UFL1 is expressed in mouse mammary epithelial cells and that its function is required for maintenance of cellular homeostasis and milk biosynthesis. Furthermore, based on the data from the UFL1-deficient *in vivo* and *in vitro* models, apoptosis was aggravated. Moreover, we revealed that UFL1 deficiency affects synthesis of milk protein and fat by suppressing JNK activation. Overall, these results provide novel insights critical to further elucidate the potential regulatory mechanisms of UFL1 depletion in the mammary gland on cellular processes.

It has been previously reported that UFL1 is expressed in different tissues. Further, UFL1 localization was observed in breast cancer, which inhibited cancer growth by interacting with UFBP1 and thus promoting UFMylation of activating signal cointegrator 1 [11]. UFL1 is also expressed in Hela and HepG2 cells, particularly within the cytosol and endoplasmic reticulum of these cells [23]. However, this is the first study to report UFL1 expression in mouse mammary epithelial cells. The mammary gland is a secretory organ that provides nutrient-rich milk to neonatal offspring and is primarily comprised of mammary epithelial cells, which include mammary alveolar epithelial cells critical for milk synthesis, indicating that UFL1 expression in these cells likely affects life processes in mouse mammary epithelial cells.

One of the most frequently occurring programmed cell death processes is apoptosis. The highly conserved caspase 3 initiates apoptosis, whereas Bcl-2, an anti-apoptotic protein, and response by mitochondrial outer membrane pore formation inhibit this process. Mounting evidence suggests that members of the UFM1 system induce apoptosis by altering caspase 3 protein levels [16, 24, 25]. Our western blot analysis revealed that cleaved caspase 3 and BAX protein levels increased in UFL1-deficient cells. Moreover, our flow cytometry analysis and TUNEL assay confirmed increased cellular apoptosis rates in response to UFL1 depletion. Therefore, these results, in conjunction with previous reports, suggest that UFL1 deletion aggravates apoptosis in mammary epithelial cells.

Notably, in the present study, UFL1 deletion did not affect mammary morphology. The exocrine pancreas is another secretory organ and synthesizes pancreatic digestive enzymes [5]. It was previously reported that pancreatic histomorphology and weight was not significantly altered by UFL1 deletion in the pancreas of UFL1 KO mice. Moreover, UFL1 deletion did not significantly affect pancreatic growth. Furthermore, paneth and goblet cells are critical intestinal components in that they synthesize proteins and antimicrobial peptides to maintain homeostatic microbiota. UFL1 deletion in intestinal sections dramatically reduced the numbers of paneth and goblet cells in the ileum [7]. Overall, our results, in conjunction with the results of these previous studies, suggest that UFL1 plays various roles in various cellular processes. Cyclin D1 is a key regulator of cell cycle progression. Increasing evidence suggests that Cyclin D1 level decreases in response to cell cycle arrest, affecting cell homeostasis and resulting in the termination of various cellular processes [26, 27]. In previous studies, percentages of cells in the S or G2/M phases increased, while cell proliferation decreased with a decrease in Cyclin D1 level, ultimately affecting milk protein and fat synthesis [28, 29]. In our study, loss of UFL1 induced a decrease in Cyclin D1 level and an increase in the rate of apoptosis, thereby disrupting homeostasis of mammary epithelial cells.

Essential roles of UFL1 in various cellular processes have been identified including ATM activation, oxidative stress, inflammation response, apoptosis, and autophagy [4, 7, 16]. It was previously found that UFL1 interaction with UFM1 is critical for the UFMylation of activating signal cointegrator 1 under estrogen to inhibit breast cancer growth. In addition to cancer growth, various life processes occur in the mammary gland, including synthesis of milk proteins. In the current study, we found that UFL1 plays an essential role in the synthesis of milk protein and fat. Milk protein synthesis is an essential and complex biological and physiological process that involves the regulation of multiple genes and pathways. In our *in vitro* and *in vivo* experiments, UFL1 deletion affected synthesis of milk protein and fat (triglyceride secretion, CSN2, ACACA, and FASN as indicators). Mounting evidence suggests that CSN2 serve as benchmarks of milk protein quality [30]. In our study, loss of UFL1 caused CSN2 levels to dramatically decline, suggesting that UFL1 deletion hinders synthesis of milk protein. Conversely, UFL1 deficiency increased triglyceride secretion, as well as ACACA and FASN levels. Thus, we speculate that UFL1 might play different roles in the synthesis of milk protein and fat.

Previous studies reported that ubiquitin-like proteins play a critical role in the regulation of the JNK pathway [18, 31, 32]. SP600125 is an effective JNK inhibitor, which is widely used to affect JNK affective in various cells [33–35]. In our study, SP600125, an effective JNK inhibitor, was used to inhibit the cellular JNK activity. Based on the western blot analysis results, we suggest that the concentration of 20 *μ*M of SP600125 was successful in decreasing JNK levels, likely blocking JNK activity. However, the UFL1 level was also decreased in response to SP600125 treatment. Thus, it is possible that a decrease in JNK level affected the UFL1 levels, and further studies are needed to verify whether JNK interacts with UFL1. Notably, in a previous study, the members of the UFM1 conjugation system were also affected by JNK activity. UFBP1, a highly conserved protein and member of the UFM1 system, activates p-JNK levels and thus regulates the JNK pathway in erythroid development [18]. Further, mitochondrial E3 ubiquitin ligase 1 is a mitochondrial membrane protein that directly activates the JNK pathway to regulate apoptosis [31]. In the current study, we found that UFL1 depletion decreased the *in vivo* and *in vitro* p-JNK protein levels. This result suggested that lower UFL1 expression suppressed JNK expression. We further found that HC11 cells cotreated with SP600125 and siUFL1 exhibited reduced CSN2 level, whereas ACACA and FASN levels increased. These results indicate that JNK is a key regulator of UFL1-mediated synthesis of milk protein and fat. Moreover, JNK plays various roles in mammary cellular processes. For example, it is critical for mammary gland remodeling [36]. It was reported that JNK promotes BAX and BCL2 expression to regulate apoptosis in epithelial cells [17]. Therefore, it is likely that UFL1 and JNK influence cellular homeostasis to suppress the synthesis of milk proteins, as observed in the present study. However, it was revealed that UFL1 deficiency induced an imbalance of endoplasmic reticulum homeostasis, as well as an increase in amylase levels in the pancreas [5]. Similarly, in our work, mammary triglyceride levels increased in response to UFL1 deficiency. Therefore, as mentioned previously, UFL1 deletion likely disrupts various cellular processes and must therefore be further studied.

In conclusion, UFL1 was expressed and played a promoter role in mammary epithelial cells by accelerating apoptotic rates and inhibiting cell viability. Moreover, UFL1 deletion affected synthesis of milk proteins and fat by suppressing JNK activation. Overall, these findings suggest that UFL1 may be a novel target for maintaining cellular homeostasis and cellular processes in the mammary gland.

## Figures and Tables

**Table 1:** Information of antibodies.

Antibodies	Cat no.	Company	Dilution of immunofluorescence	Dilution of western blot
UFL1	26087-1-AP	Proteintech Group	1 : 200	1 : 1000
BAX	14600-1-AP	Proteintech Group	_____	1 : 1000
BCL2	18420-1-AP	Proteintech Group	_____	1 : 1000
Cleaved caspase 3	#9664	Cell Signaling Technology	_____	1 : 1000
Cyclin D1	26939-1-AP	Proteintech Group	_____	1 : 1000
CSN2	A12749	ABclonal	_____	1 : 2000
ACACA	A15606	ABclonal	_____	1 : 1000
FASN	A19050	ABclonal	_____	1 : 1000
GAPDH	10494-1-AP	Proteintech Group	_____	1 : 4000
Goat-IgG Rabbit	10285-1-AP	Proteintech Group	_____	1 : 4000
Alexa Fluor 647-conjugated Goat Anti-Rabbit IgG (H+L)	AS060	ABclonal	1 : 300	_____

**Table 2:** Primer sets for quantitative real-time PCR.

Gene	GeneBank number	Primer	Sequence of nucleotide (5′-3′)
*UFL1*	NM_001355512	F	GTTGACATTTCGCCTCTGCT
		R	TCACCACAACCGTGTCACTA

*GAPDH*	NM_001034034.2	F	AACGGATTTGGCCGTATTGG
		R	CATTCTCGGCCTTGACTGTG

## Data Availability

The data used to support the findings of this study are available from the corresponding author upon request.

## Supplementary Materials

Supplementary Materials Supplement Figure 1: Effect of UFL1 decreased on cell proliferation in mouse mammary. (A) The expresssion of Cyclin D1 was detected by western blot in the mammary gland of UFL1 KO mice. The relative intensity of A was plotted in B. (C and D) After HC11 cells were transfected by siUFL1 and control siRNA, the level of UFL1 were analyzed by western blot. Different letters indicate significant difference (*P* < 0.05).
