# Supplementary material for: UFM1-Specific Ligase 1 Ligating Enzyme 1 Mediates Milk Protein and Fat Synthesis-Related Gene Expression via the JNK Signaling Pathway in Mouse Mammary Epithelial Cells
Source: Oxid Med Cell Longev. 2020 Jun 19;2020:4045674. doi: 10.1155/2020/4045674 (PMC7321527; doi:10.1155/2020/4045674)
Supplement: Supplementary Materials — Supplement Figure 1: Effect of UFL1 decreased on cell proliferation in mouse mammary. (A) The expresssion of Cyclin D1 was detected by western blot in the mammary gland of UFL1 KO mice. The relative intensity of A was plotted in B. (C and D) After HC11 cells were transfected by siUFL1 and control siRNA, the level of UFL1 were analyzed by western blot. Different letters indicate significant difference (P < 0.05). [file 4045674.f1.pdf]

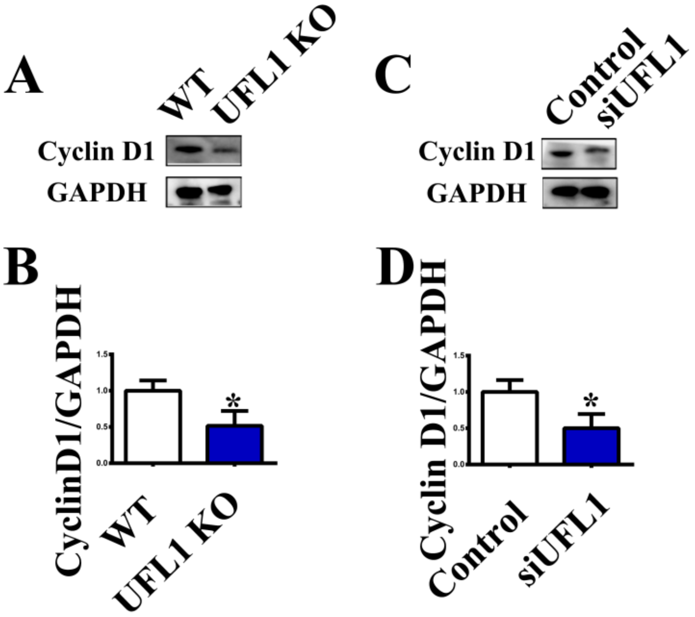


**Supplement Fig 1**

Supplement Fig 1. Effect of UFL1 decreased on cell proliferation in mouse mammary. (A) The expression of CyclinD1 were detected by western blot in mammary gland of UFL1 KO mice. The relative intensity of A was plotted in B. (C and D) After HC11 cells were transfected by siUFL1 and control siRNA, the level of UFL1 were analyzed by western blot. Different letters indicate significant difference (*P*<0.05).
